# The patient experience of Wilson disease: a conceptual model based on qualitative research

**DOI:** 10.1186/s13023-021-02059-x

**Published:** 2021-10-19

**Authors:** Stella Karantzoulis, Karli Heuer, Nicole Sparling, Megan Teynor

**Affiliations:** 1 IQVIA, 300 Vesey Street, 13th Floor, New York, NY 10282 USA; 2 Alexion Pharmaceuticals Inc., 121 Seaport Blvd, Boston, MA 02210 USA

**Keywords:** Patient interview, Concept elicitation, Hepatolenticular degeneration, Symptom presentations, Disease impacts

## Abstract

**Background:**

Wilson disease (WD) is a rare disease wherein copper accumulates in tissues, leading to hepatic degeneration, neurological impairments, and psychiatric symptoms. This study aimed to characterize the patient experience of WD and develop a conceptual model containing key symptoms and impacts of the disease.

**Results:**

A targeted literature review was conducted to develop a preliminary conceptual model of WD that was subsequently refined through one-on-one interviews with 3 WD clinicians and finalized following concept elicitation interviews with 11 patients and 1 caregiver. The literature review returned 30 articles, from which 45 concepts (35 signs/symptoms and 10 impacts) were selected for inclusion in the preliminary conceptual model. After interviews with clinicians, the model was expanded to include 45 signs/symptoms and 14 impacts. The final comprehensive conceptual model developed after interviews with patients included 54 symptoms in total (n = 22 hepatic, n = 19 neurological, n = 13 psychiatric), and 21 impacts. Across symptoms, patients reported a high level of bother, with approximately 49% of symptoms reported by patients having an average peak bother rating of ≥ 7 out of 10 (10 = most bothersome). Patient interviews identified 2 subgroups of patients: those who experience neurological, psychiatric, and hepatic symptoms and those who experience mostly hepatic and some psychiatric symptoms, but no neurological symptoms.

**Conclusions:**

This research underscores the substantial multisystemic symptoms and impacts that patients with WD describe as highly bothersome in their lives. Hepatic symptoms emerged as especially common and important to patients with WD, possibly beyond what is commonly understood in research and clinical practice. Further, the description of 2 distinct patient groups may help to inform patient management and support more targeted drug development processes.

**Supplementary Information:**

The online version contains supplementary material available at 10.1186/s13023-021-02059-x.

## Background

Wilson disease (WD) is an inborn autosomal recessive disorder of impaired copper (Cu) transport. Over time, Cu accumulates in liver, brain and other tissues, resulting in progressive organ damage and dysfunction that can vary from patient to patient [1]. Diagnosis can occur in childhood, but in some cases may be later in life [2]. Clinical prevalence estimates for WD range from approximately 1 per 30,000 to 1.5 per 100,000 worldwide [3–5] although the frequency of genetic markers for WD may be higher in selected regions such as in some Asian communities, where geographic isolation has given rise to increased prevalence due to consanguineous transmission [2, 6]. Among people with an identified mutation, disease manifestation will be present in approximately 50% of individuals [4, 7].

Management of WD often requires a multidisciplinary approach that combines hepatology, neurology and psychiatry specialists, given the potential involvement of all 3 systems in the clinical presentation of patients with WD [1, 8–11]. Currently available therapies for the treatment of WD include the general chelation therapies D-penicillamine and trientine, which non-specifically chelate Cu and promote urinary Cu excretion. In addition, zinc (Zn), which blocks uptake of dietary Cu, is used for maintenance treatment. These therapies need to be dosed 2 to 5 times per day and should be taken in the fasted state. These therapies also have high rates of treatment discontinuation due to poor tolerance [12, 13]. Common adverse events include gastric discomfort, anemia, elevated liver function tests, proteinuria, autoimmune disorders, bone marrow suppression, and neurological worsening [14]. Although liver transplant is an option for patients with predominantly hepatic manifestations, its use is debated in patients with WD and progressive neurological deterioration. Regardless of presentation, patients with WD require life-long therapies [15].

The impact of WD to patient’s health-related quality of life (HRQoL) has not been extensively assessed. However, the few published studies focusing on the humanistic burden of WD show the negative impact of the disease on HRQoL of patients and their caregivers. Patients with WD are at risk of depression [16, 17] and have been reported to have low HRQoL as assessed with the 36-Item Short Form Health Survey (SF-36), particularly when patients have primarily neurological or psychiatric manifestations (for instance, anxiety and depression) [17, 18]. Increased psychiatric symptoms such as depression and anxiety have a substantial impact on patient quality of life. Other psychiatric and neurological features of the disease, including emotional and behavioral dyscontrol, limit the ability of patients to participate in socialization and other daily activities (16). HRQoL is worse among patients with decreased functional mobility and limited ability to interact within their environment and society [18]. In patients with WD, the physical domain of the World Health Organization Abbreviated Quality of Life measure (WHOQoL-BREF) has been shown to be inversely correlated with treatment duration and disease severity [16]. In addition, patients with WD scored significantly worse on the generic 12-Item Short Form Health Survey (SF-12) tool when compared to controls without WD selected from an Italian epidemiological database used to study health conditions [16]. Sex differences in WD have also emerged that may affect HRQoL as women with WD report significantly lower HRQoL scores than men [17].

The aim of this study was to characterize the experience of patients aged 12 years and older with WD, and to develop a conceptual model representing the key symptoms and impacts of WD. Clarifying the patient perspective of WD can then be used to guide conversations between patients and clinicians with the goal of improving disease management, as well as to inform drug development and improve selection of trial endpoints to better meet the needs of patients with WD.

## Methods

The study was conducted in several steps. A targeted literature review (TLR) was first conducted to develop a preliminary conceptual model of WD. This model was refined based on the findings from one-on-one telephone interviews with clinicians actively treating people with WD and was then finalized following input from one-on-one telephone interviews with patients and a single caregiver.

### Literature review

A TLR was first conducted in April 2019 to identify publications describing the patient experience of WD in terms of signs (clinical markers of a disease, often determined by a physician), symptoms (features of a disease experienced and reported by a patient), and impacts (effects of a disease upon a patient’s daily function and wellbeing) in adolescent (aged ≥ 12 years) and adult patients with WD. Searches were conducted in PubMed and Google Scholar. Key search terms included “Wilson’s Disease” and any one of the following terms: symptoms, signs, manifestations, impacts, PROs [patient-reported outcomes], COAs [clinical outcome assessments], UWDRS [Unified Wilson's Disease Rating Scale], and “rating scale”. Articles were excluded if (1) they had been published 10 or more years ago, (2) the study focused on children, (3) the study was a non-human trial, or (4) the article was not related to signs, symptoms, impacts, or patient HRQoL. Articles beyond 10 years were excluded to prioritize the most current understanding of the patient presentation of WD.

Based on the prior understanding of the disease presentation, identified signs, and symptoms (i.e., concepts) were organized into the 3 major categories: neurological, psychiatric, and hepatic. Likewise, impacts were also organized as immediate impacts (those proximal to the associated symptoms of WD) and general impacts (overall effects that may be related to immediate impacts). Concepts identified in the literature were used to create a preliminary conceptual model. Concepts were prioritized if the higher end of the prevalence range stated in the literature was ≥ 50%. Additionally, social media listening, or the extraction of quantifiable data from online channels such as social networking sites, blogs, and forums, was conducted to identify any additional concepts that are meaningful to patients. The resulting concepts from the preliminary conceptual model and the social media listening were discussed with clinicians during their interviews.

### Clinician interviews

One-on-one in-depth qualitative telephone interviews were conducted with 3 expert clinicians recruited based on their practice area and experience in the management of patients across the lifespan with WD. One clinician was interviewed within each relevant subspecialty—neurology, psychiatry, and hepatology. Additional selection criteria included having a clinical practice in the US (with > 10 years’ experience) and frequently seeing patients in their practice and managing their WD care.

Interviews were conducted over the telephone and lasted approximately 60–75 min. Interviews were led by a trained interviewer, and a semi-structured discussion guide was used to facilitate the conversations. Clinicians were first asked to discuss, without prompting, the signs, symptoms and impacts patients and their caregivers report, and then any additional signs, symptoms and impacts that they observe. After unprompted discussion, clinicians were probed on any remaining concepts from the preliminary conceptual model, or concepts that had been identified through social media listening. Interviews were recorded and subsequently transcribed for analysis. Insights from clinician interviews were used to revise the preliminary model to an interim conceptual model.

### Patient and caregiver interviews

Interviews were conducted in accordance with the 1975 Declaration of Helsinki and the regulations of the US Food and Drug Administration. The study and all interview materials received a priori Independent Review Board (IRB) approval.

Semi-structured concept elicitation (CE) interviews were conducted with 11 patients with WD and one caregiver of one of the patients included in the interviews (a patient diagnosed at age 15 who was 22 years old at the time of the interview). Patients were recruited from the US (n = 8), Germany (n = 2), and the UK (n = 1) via 2 separate recruiting services, Global Perspectives (US and Germany) and Medicys Ltd (UK), and through Patient Advocacy Groups (US). Screening criteria was set such that participating patients had to have a clinical diagnosis of WD and be 12 years of age or older in the US and 18 years of age or older in the European Union. Patients also had to report experiencing symptoms due to WD or the treatment they were receiving for WD during the last 4 months. Additional details on the selection criteria are provided in the Additional file 1: Table S3.

All participants completed the screener online or over the telephone to determine their eligibility and provided their consent before the start of the interview. Patients’ clinicians or clinicians’ offices completed a confirmation of diagnosis form that asked for information regarding diagnosis of WD, diagnosis of liver failure, diagnosis of end-stage renal disease or patient use of dialysis, and treatment history with ammonium tetrathiomolybdate.

All interviews in the US and the UK were conducted in English by a single licensed clinical psychologist with expertise in qualitative interviewing. The interviews in Germany were conducted by a local trained interviewer in German. Interviews were conducted over the telephone and lasted approximately 90 min (ranging from 51 to 120 min) for patients and 60 min for the caregiver. An IRB-approved semi-structured interview guide was used to guide the conversation and ensure consistency in the interview content.

The interviews were structured in 3 sections and were designed to last approximately 90 min. The first section focused on general demographic questions. The second section explored the patient journey with WD, including time since diagnosis and treatment history. The third section focused on the patient experience with WD (signs, symptoms, and impacts). Signs, symptoms and impacts of WD, currently and previously experienced, were first obtained unprompted, or spontaneously, followed by more prompted exploration of signs, symptoms and impacts using probes for discussing those concepts that had not yet been mentioned by the patient but which had been previously identified from the TLR and clinician interviews. For each concept currently experienced, patients were asked to provide a current peak bother rating on a scale from 0 (not at all bothered) to 10 (greatly bothered). For each concept previously experienced, patients were asked to provide a past peak bother rating using the same scale. Ratings were used to help quantify the effect of each concept throughout the patient’s disease history.

Over the course of the patient interviews, signs/symptoms and impacts were discussed. The full concept list was based on input from several sources and evolved over time as new concepts were mentioned and refined. Specifically, the full concept list was informed by the literature review, social media listening, and clinician interviews described above as part of this study. It was also initially informed by a separate CE patient interview study with WD patients who were recruited specifically because they have a history of neurological signs/symptoms. These separate CE interviews were initiated just prior to the interviews described in the current study, and patients were recruited via the same channels with the only difference being that the WD patients reported a history of experiencing neurological signs/symptoms to qualify for the study [19].

The CE interviews for the current study were conducted in 3 waves (Wave 1, n = 4; Wave 2, n = 4; Wave 3, n = 3). Patient transcripts were analyzed chronologically after each wave to assess whether concept saturation had been reached. Saturation of concepts was defined as the point at which no new information was forthcoming from ensuing patient interviews [19]. Saturation of concepts was determined to be adequate after the third wave of interviews (i.e. after 11 interviews).

### Data analysis

Interviews were audio-recorded and subsequently transcribed for analysis using ATLAS.ti v8 qualitative analysis software. Coders reviewed the transcripts to identify relevant concepts by tagging the relevant text with a code. Codes were then organized within a coding framework, which had been established before coding started and was refined during the coding process. Two coders were involved in the coding of the transcripts, and inter-rater agreement (IRA) was evaluated to be maintained above ≥ 0.7.

Qualitative research methods were used to determine the most salient concepts based on the number of patients who experience each and the average peak bother reported. Concepts were considered salient if they were reported by ≥ 5 patients (i.e., ≥ 5 patients report ever experiencing the concept regardless of whether it was experienced in the past, current, or both) and the average peak bother rating for the concept was ≥ 5. The average peak bother represents the average of the highest reported bother rating from each patient regardless of timepoint (i.e., if the bother rating was reported in the past or current). Due to the open-ended, semi-structured nature of the interviews, patients did not necessarily rate every concept that they reported. Additionally, not every concept was discussed with every patient, as some concepts did not arise until later waves (Additional file 1: Tables S4 and S5). Patients who did not provide a peak bother rating were not included in any of the average peak bother rating calculations and also not factored into determining the number of patients ever experiencing each concept. The data from the patient interviews was synthesized to develop the final conceptual model of WD. Data were also analyzed to explore the change in the patient experience over time and to evaluate potential subgroups of patients based on symptoms reported.

A posthoc analysis was conducted to ensure that comorbidities did not substantially influence the concepts being identified as important to WD. To achieve this, patients with comorbidities were removed (one at a time) from the analysis of salient concepts, and the changes in number of mentions and/or bother ratings was assessed.

## Results

### Targeted literature review

The TLR identified 569 articles of which 30 were included (Additional file 1: Table S1). From these 30 articles, 45 concepts (35 signs/symptoms and 10 impacts) associated with WD were identified for inclusion in the preliminary conceptual model.

Priority concepts (those where the upper prevalence range was ≥ 50% in the reviewed articles) included both neurological (such as slurred speech/speech disturbances, gait abnormalities, parkinsonism, tremors) and psychiatric (such as increased irritability/anger outburst, disinhibition) symptoms but no hepatic symptoms. Only one impact (catatonic/abnormal movement) also met the criteria for a prioritized concept. All concepts retrieved from the literature review were included in the preliminary WD conceptual model, with prioritized concepts in bold (Additional file 1: Fig. S1).

### Clinician interviews

The clinicians interviewed were leading experts in the study of WD, had between 12 and 32 years of clinical experience, and were currently practicing in academic medical centers. Each clinician had a different clinical subspecialty that aligned with the 3 core symptom categories (neurological, psychiatric, and hepatic; Additional file 1: Table S2).

Clinicians broadly agreed with the preliminary conceptual model. Ten of the signs/symptoms from the preliminary conceptual model were confirmed by clinicians as relevant to the patient experience and were maintained in the updated model with no changes to the wording as they were considered appropriately patient-friendly. These 10 signs/symptoms were tremor, headache, difficulty swallowing, yellow skin (jaundice), tendency to bleed easily, seizures, vomiting, fainting, joint pain, and joint stiffness. Table 1 describes changes made to signs and symptoms following clinician interviews. Clinicians recommended that the wording of 18 of the remaining signs/symptoms be revised to be made more patient-friendly; the language was amended with guidance from an experienced interviewer and qualitative researcher. Three signs and symptoms were removed from the conceptual model because they were considered to be treatment-related or not associated with the natural history of WD. Clinicians considered 6 additional symptoms identified through social media listening as relevant and proposed to include them in the conceptual model. In addition, clinicians mentioned 2 new signs/symptoms that were also added to the model.

**Table 1:** Changes based on clinician interviews

Signs/symptoms modified		Impacts modified	
Changes to existing symptoms		Changes to existing impacts	
Preliminary model	Revised	Preliminary model	Revised
Gait abnormalities	Changes in walking	Planning difficulties	Difficulty planning
Salivation	Drooling	Suicide attempts	Intentional self-harm
Bipolar disorder/mania	Mania	Self-injurious behavior	
Cognitive impairment	Changes in thinking skills (e.g., feeling slowed down, forgetful)	Catatonic/abnormal movement	Abnormal body movements
Attention deficit	Changes in attention (e.g., trouble focusing, easily distracted)	Limitations in function/daily activities	Limitations in physical function
Emotional lability	Frequent “ups and downs” in mood	Increased irritability/anger outburst	Anger outburst
Increased irritability	Irritability	Sleep disturbances/excessive daytime sleeping	Sleep disturbances
Apathy	Apathy (e.g., feeling disengaged, feeling like you do not care about anything anymore)		Excessive daytime sleeping
Hyperactivity	Hyperactivity (e.g., cannot sit still, restless)		
Psychosis	Psychotic episode (e.g., hearing voices that no one else hears, seeing things that are not really there)		
Abdominal pain	Stomach pain		
Spider veins	Spider veins (i.e., small, damaged veins visible on the surface of the legs or face)		
Frailty	Frail (e.g., fragile, physically vulnerable / weak)		
Fatigue	Fatigue (e.g., extreme tiredness, low energy levels)		
Swelling	Swelling/fluid retention		
Slurred speech/speech disturbances	Slurred speech		
	Other changes in speech (e.g., vocal tremor, stuttering, slow speech)		
Parkinsonism	Changes in balance		
	Changes in facial expression		
Dysexecutive syndrome	Difficulty solving problems		
	Difficulty with decision making		

Symptoms removed	Impacts removed
Hair loss	Catatonia
Dry skin	Anger outbursts
Hypertension	Sudden physical collapse following strong emotion
	Dementia
	Anorexia

Symptoms added	Impacts added
Night sweats	Difficulty writing
Dizziness	Inability to walk/wheelchair bound
Shortness of breath	Change in work performance
Anaemia	Change in school performance
Enlarged/swollen liver	Impact on family life
Numbness in jaw	Impact on social life
Vertigo	
Kayser–Fleischer rings (greenish brown or golden rings around your eyes)	

Table 1 also describes changes made to impacts of WD on daily patient life in the model following clinician interviews. Overall, the wording for 7 impacts was revised to render them more patient-friendly, 5 were excluded from the revised interim conceptual model because they were not considered relevant to the WD patient experience, and 6 new impacts were added. The interim conceptual model includes 45 signs/symptoms and 14 impacts (Additional file 1: Fig. S2).

### Patient CE interviews

#### Patient sample demographics and clinical history

Between February 2020 and June 2020, CE interviews were conducted with 11 patients with WD from the US (n = 8), Germany (n = 2), and UK (n = 1; Table 2). All but one patient was female (n = 10), and patients ranged in age from 18 to 65 years (mean = 38.9). Patients were diagnosed with WD between 5 and 55 years ago. Over the course of their disease history, these patients received a variety of treatments, both prescription and non-prescription. All patients reported having adhered to a low Cu diet at some point during their diagnostic history. All patients were receiving treatment, whether prescription or dietary, at the time of the interview.

**Table 2:** Patient baseline demographics and clinical history

Characteristic	N
Total sample*	11
Age at time of study	
Under 18	0
18–29	4
30–39	1
40–49	4
50–59	1
60–69	1
70+	0
Gender	
Male	1
Female	10
Educational status, highest level attained	
High school	4
College	5
Graduate degree	2
Geography	
United States	8
United Kingdom	1
Germany	2
Years since diagnosis	
0–5	2
6–10	2
11–20	5
20+	2
Treatment status	
Currently receiving treatment (prescription or non-prescription)	11
Not receiving treatment	0
Treatment (ever received)	
Trientine	7
Penicillamine	4
Zinc	6
Low copper diet	11
Other**	3

^*^10/11 confirmation of diagnosis forms received

^**^Occupational and physical therapy, supplements

Five of the interviewed patients presented with substantial comorbidities (Table 3).

**Table 3:** Patients with significant comorbidities

Patient	Comorbidities
Patient 2	Chronic lymphocytic leukemia (CLL)
	Arthritis
	Gallbladder removal
	Lupus
Patient 6	Migraines
	Fibromyalgia
	Post-traumatic stress disorder (PTSD)
	Kidney stones
Patient 8	Narcolepsy
	Gallbladder removal
Patient 9	Hashimoto’s disease
	Migraines
	Polycystic ovary syndrome (PCOS)
	Dysmetria
	Left-sided weakness
Patient 11	Fatty liver disease
	Degenerative joint disease
	Asthma
	Adenomyosis
	Hearing impairment
	Hashimoto’s disease
	Migraines

#### Patient and caregiver experience of WD

During the patient interviews, 74 signs/symptoms and 23 impacts were included for discussion.

##### Signs and symptoms

Across patient interviews, 63 different signs/symptoms were reported. Of these, 31 were considered hepatic, 19 neurological and 13 psychiatric. Patients reported an average of 24 different signs/symptoms over the course of their WD and specified experiencing an average of 21 signs/symptoms at the time of the interviews.

All but 3 symptoms were mentioned by patients in Wave 1 (n = 4) or Wave 2 (n = 4; Additional file 1: Table S4). Of the 3 additional symptoms, vertigo and slurred speech were reported by the first patient in Wave 3 (n = 3), and fainting was mentioned by the second patient in Wave 3. As only 3 new concepts were described in Wave 3, and the last interviewed patient did not raise any new concepts, it was concluded that saturation for signs/symptoms was adequate.

**Fig. 1:**
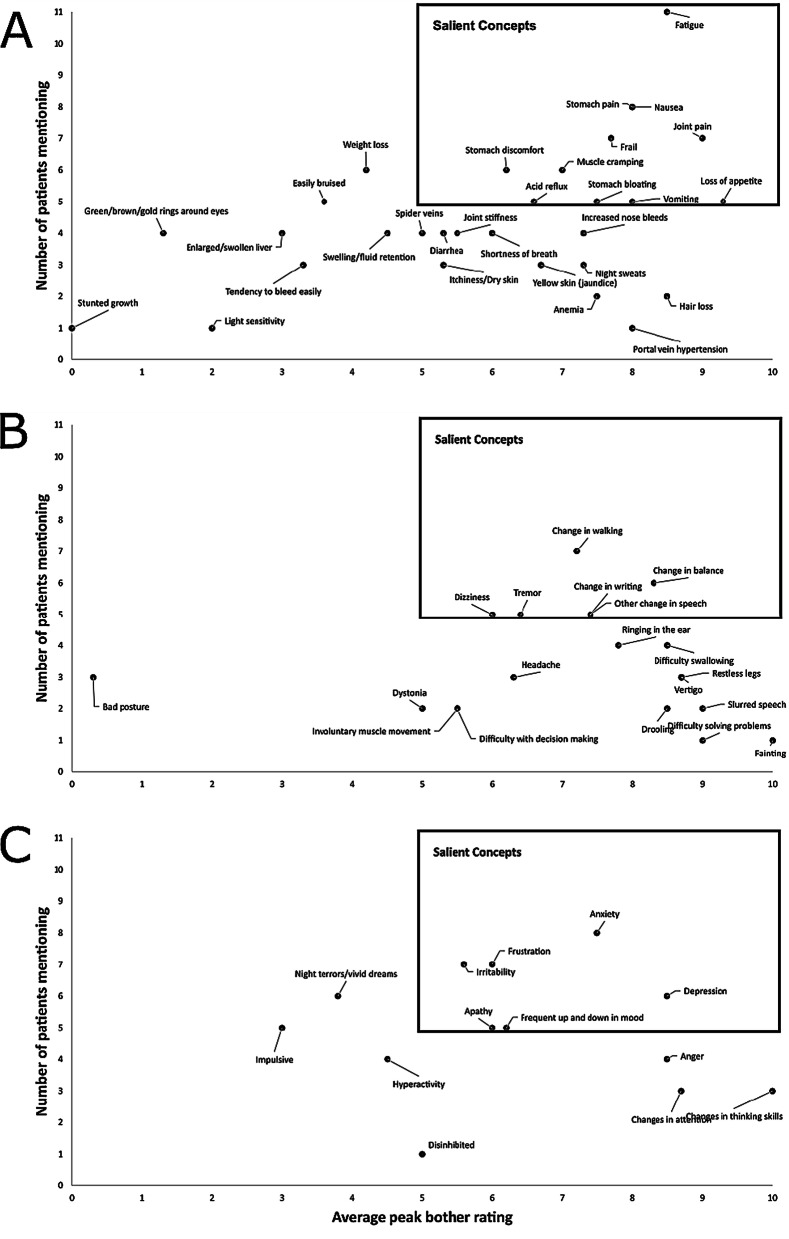
Average peak symptom bother and number of mentions. **a** Hepatic, **b** neurological, **c** psychiatric. Y-axis represents the number of patients ever reporting experiencing the concept (either currently or in the past)

Several hepatic (6), neurological (6), and psychiatric (11) signs and symptoms were considered salient (defined as ≥ 5 people ever experiencing a concept and the average peak bother rating ≥ 5). Among hepatic signs/symptoms, fatigue and nausea were the most commonly reported by patients, and both were considered highly bothersome (average peak bother ratings of 8.5 and 8.0, respectively; Fig. 1, Additional file 1: Table S6). Other salient hepatic symptoms included stomach pain (8), frail (7.7), joint pain (9), stomach discomfort (6.2), muscle cramping (7), vomiting (8), stomach bloating (7.5), loss of appetite (7.6), and acid reflux (6.6).

Hepatic symptoms featured prominently in patients’ description of their disease:

I feel like I have phases where I’m good and I’m ready to go. Then sometimes I’m like I’m just so tired, I feel like have nothing…even though I have a full night of sleep (Patient 4).

I’m always tired. It’s one of those things where you wake up in the morning and you think you’re going to be rejuvenated from the night before if you sleep. I do have trouble sleeping, also. I’m just tired. I could sit down, but I can’t…Kind of like somebody hit me with a truck (Patient 7).

I was constantly nauseous. I rarely vomited, but me and food did not get along (Patient 9).

[Nausea] That was a full-blown 10+…I couldn’t even get up in the mornings (Patient 10).

Among neurological signs/symptoms reported, change in walking (7.2), change in balance (8.3), tremor (6.4), other changes in speech (7.4), change in writing (7.4), and dizziness (6) can be described as salient.

Patients regularly described their difficulty with walking, balance and other neurological symptoms:

Sometimes it takes me a minute to recall the words that I want. Sometimes I actually say weird things because I’m trying to find my words, but the problem is I can’t, so I have to use words around it (Patient 4).

This is not like severe shaking. But it is certainly uncomfortable, because I cannot grip properly or hold on to things (Patient 5).

So, walking gets difficult because of changes in balance. So, my gait pattern changes because of that (Patient 5).

Dragging my feet mostly. Dragging my feet, and then sometimes, I’ll just try and take a step and then it feels almost like I’m dizzy and I have to catch myself (Patient 6).

I kind of sway back and forth sometimes walking…Like I can’t walk in a straight line (Patient 9).

Among psychiatric signs/symptoms, anxiety (7.5), irritability (5.6), frustration (6), depression (8.5), frequent up and down in mood (6.2), and apathy (6) were considered salient.

Patients detailed their experience of how WD was associated with a variety of psychiatric symptoms:

I am very irritated easily. The littlest things just set me off. I get frustrated and impatient…I would say 9–10 because typically I’m a really relaxed calm easy-going person (Patient 1).

I’ve always had mood swings, but they weren’t really pronounced. I noticed them, but other people never noticed them (Patient 9).

It’s [anxiety] pretty much my whole life. Within the past few years it’s been more than ever (Patient 11).

Seventy-nine percent of all of the reported symptoms had an average peak bother rating of ≥ 5, while approximately 49% of symptoms had an average peak bother rating of ≥ 7.

The following symptoms are not depicted due to not having been mentioned by patients: changes in facial expression, asymmetry of face, unable to walk/unable to talk, difficulty eating, seizures, numbness in jaw, psychotic episode, mania, lower extremity pain related to swelling, skin rash, blurred vision. In addition, joint swelling was mentioned by 1 patient but was not probed for a bother rating.

All impacts were mentioned by patients in Wave 1 except limitations in usual daily activity, which was mentioned in wave 3, despite being probed with all patients (Additional file 1: Table S5). As only 1 new concept was described in the final wave of interviews, saturation of impacts was determined to be adequate.

**Fig. 2:**
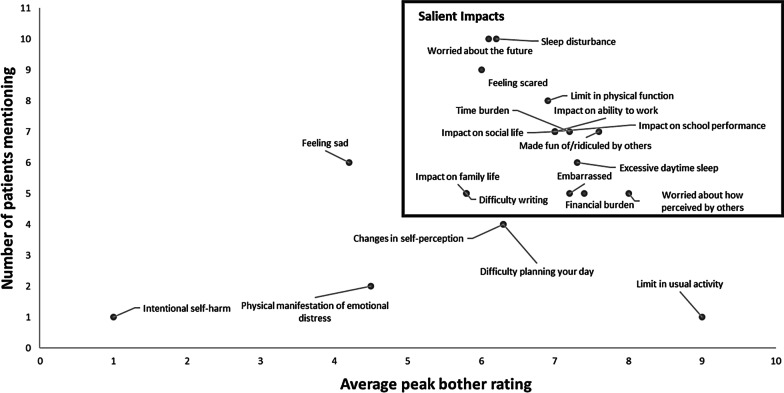
Average peak impact bother and number of mentions. Y-axis represents the number of patients ever reporting experiencing the concept (either currently or in the past). The following impacts are not depicted due to not having been probed for bothersome ratings: Inability to walk/wheelchair bound, and Alcohol/abuse. The following impacts are not depicted due to having not been mentioned by patients: Seizures, and Numbness in jaw

Fifteen impacts were found to be most salient to patients: sleep disturbance (average peak bother 6.2), worried about the future (6.1), feeling scared (6), limit in physical function (6.9), impact on social life (7), impact on ability to work (7), impact on school performance (7), made fun of/ridiculed by others (7.6), excessive daytime sleep (7.3), time burden (7.2), financial burden (7.4), embarrassed (7.2), worried about how perceived by others (8), difficulty writing (5.8), and impact on family life (5.8; Fig. 2; Table S7 in the Supplemental information).

Patients described in detail the varied and profound effect that WD symptoms had on their daily lives and HRQoL:

Well, like I had mentioned I’m scared to the point where if I do ever have to have a liver transplant because having surgery, and having to be on a liver transplant list, and things like that, that’s scary (Patient 1).

When I was diagnosed, it was like, don’t play with XXX, she’s got a disease. Don’t go near XXX, you’ll capture it (Patient 2).

A lot to do with being tired and pain. So, I think I have a lot of physical limitations consistently. So, I’d give that a 6 (Patient 6).

And, my social life too. I feel like I’m going to sit there and nod out. Can I go out to dinner? Once in a while. Can I drink? Previously, obviously. Go to a friend’s house and there I am sitting in a chair and sleeping, because I’m just so exhausted (Patient 7).

…I got to the point where I would fall asleep in class. My teacher would wake me up. I would go to the next class. I would fall asleep and so forth until the rest of the day, and then I’d get home I could fall asleep (Patient 8).

It actually took me eight years to get my degree because of my fatigue mostly (Patient 11).

I wonder if I can ever have a full-time career…So I often worry about what I can do with my life (Patient 11).

The single caregiver interview confirmed the findings of the patient interviews in terms of experience with WD and key signs, symptoms, and impacts. Additional insights from the caregiver focused on the burden of the financial impact the current treatment had on the family.

#### Patient experience with comorbidities

Comorbidities (Table 3) were not found to have a substantial impact on the WD patient experience. The same concepts were elicited by patients with and without comorbidities but there were some differences in salience when patients with comorbidities were removed one at a time from the analysis. Specifically, certain neurological (other changes in speech, tremor, change in writing and dizziness), hepatic (vomiting, stomach bloating and acid reflux), and psychiatric symptoms (apathy and frequent up and down in mood) were less salient when patients with comorbidities were removed from the overall analysis of salient symptoms. In contrast, other symptoms such as night terrors/vivid dreams (psychiatric) and weight loss (hepatic) became relatively more salient when patients with comorbidities were removed from the sample. The only difference with respect to impacts was that feeling embarrassed, worried about how they are perceived by others, impact on family life and difficuly writing became less salient.

#### Patient subgroups

Qualitative CE patient interviews identified 2 subgroups of patients with WD based on the symptoms they experienced (Table 4). One group consisted of patients who experience a range of neurological, psychiatric, and hepatic symptoms (7 patients). The other group consisted of patients who experience mostly hepatic and some psychiatric symptoms (4 patients) but no neurological symptoms.

**Table 4:** Symptoms experienced by patients

	Wave 1				Wave 2				Wave 3		
	P1	P2	P3	P4	P5	P6	P7	P8	P9	P10	P11
Total number of neurological symptoms	0	0	6	10	7	9	0	0	13	12	8
Total number of psychiatric symptoms	2	6	6	8	7	10	0	2	11	5	7
Total number of hepatic symptoms	7	22	9	13	15	18	7	12	15	13	7
Total	9	28	21	31	29	37	7	14	39	30	22
Neurological load	0%	0%	**29%**	**32%**	**24%**	**24%**	0%	0%	**33%**	**40%**	**36%**
Psychiatric load	22%	21%	**29%**	**26%**	**24%**	**27%**	0%	14%	**28%**	**17%**	**32%**
Hepatic load	78%	79%	**42%**	**42%**	**52%**	**49%**	100%	86%	**39%**	**43%**	**32%**

Cells that are not bolded represent patient group 1 (hepatic and some psychiatric symptoms); bolded cells represent patient group 2 (neurological, psychiatric, and hepatic symptoms)

For those patients who report all 3 symptom categories (7/11), almost one-third (31%) of their total symptoms were neurological, while 26% were psychiatric and 43% hepatic. For those patients who experience primarily hepatic symptoms and some psychiatric symptoms, (4/11), 14% of their total symptoms were psychiatric and 86% hepatic.

For both patient groups, the symptoms experienced were highly bothersome. For those patients who had all 3 categories of symptoms, 66%, 77% and 95% of the hepatic, psychiatric and neurologic symptoms, respectively, had an average bother rating ≥ 5. For patients with WD with primarily hepatic and some psychiatric symptoms, 73% of hepatic symptoms and 71% of psychiatric symptoms had an average bother rating ≥ 5.

#### Final conceptual model

**Fig. 3:**
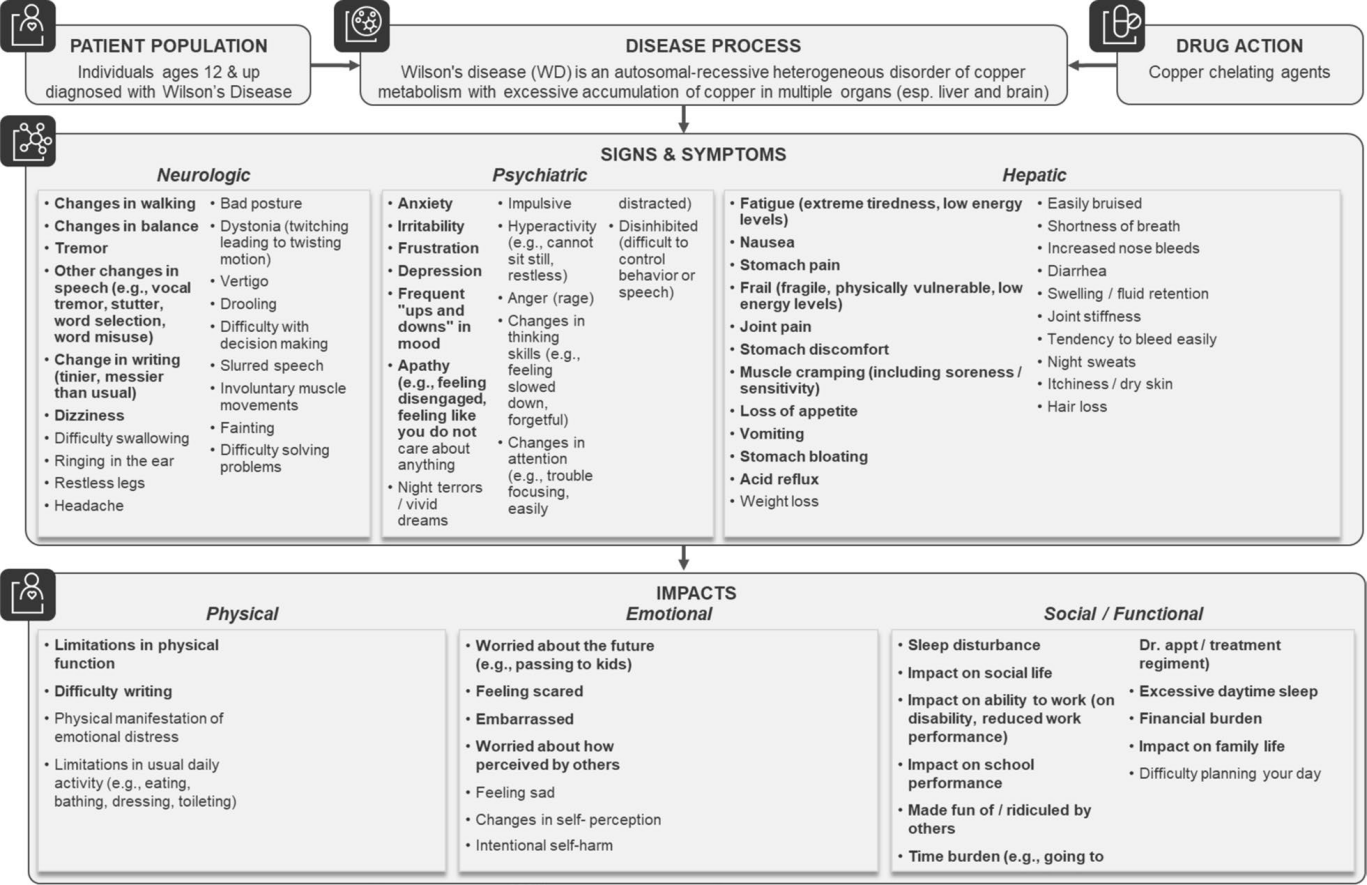
Final WD conceptual model

The final conceptual model includes 54 symptoms (22 hepatic, 19 neurological, 13 psychiatric) and 21 impacts with salient concepts depicted in bold (Fig. 3).

Twenty of the 74 symptoms discussed with patients were excluded from the final conceptual model because they were not endorsed by patients (unable to walk/unable to talk, difficulty eating, numbness in jaw, lower extremity pain related to swelling, skin rash, blurred vision), they would unlikely change with treatment in a clinical trial (changes in facial expression, asymmetry of face, stunted growth, spider veins), or they would not be easily captured through PRO measures (seizures, psychotic episode, mania, enlarged/swollen liver, anemia, portal vein hypertension, yellow skin [jaundice], green brown/gold rings around the eyes). In addition, joint swelling was removed due to low number of mentions and light sensitivity was removed because it was raised in the context of reactivity of the patient’s blue eyes to light (e.g., during routine eye exams) and was not considered related to the disease.

Although hair loss and dry skin had previously been excluded from the interim conceptual model based on clinician interviews, patients mentioned these symptoms and considered them at least partly related to the disease. Itchiness and dry skin were merged into a single concept (itchiness/dry skin) and were included in the final model.

Of the 23 impacts discussed with patients, the final model excluded inability to walk/wheelchair bound, and alcohol/abuse because they were not reported by patients in the interviews. Impacts were reorganized from immediate and general categories into 3 conceptual categories to better reflect feedback from interviews: physical, emotional and social/functional (Fig. 3).

## Discussion

The results of this research have generated a conceptual model of the symptomatic patient experience with WD that highlights the substantial bother associated with patients who experience these symptoms as well as the marked impact these symptoms have on the lives of patients with WD. Some patients with WD may be asymptomatic, however, the purpose of this study was to better understand the signs, symptoms and impacts that patients with WD experience. Therefore, patients who were not symptomatic in the past 4 months were not included in this interview study. The resulting conceptual model applies to symptomatic WD population.

Individuals with WD experience many highly bothersome symptoms that span across multiple bodily systems, resulting in hepatic, neurological, and psychiatric impairments. Among the 11 patients with WD who participated in CE interviews in this study, patients reported having experienced, on average, 24 different signs/symptoms over the course of their disease (5–55 years since diagnosis) and 21 signs/symptoms, on average, at the time of the interviews. Importantly, patients reported a high degree of bother associated with the symptoms, with almost 80% of the symptoms having an average peak bother rating of 5 out of 10 or more, while nearly half of symptoms had an average peak bother rating of 7 or more. This high humanistic burden remained in evidence even when patients with substantial comorbidities were excluded from the analyses.

There were many salient symptoms across all 3 symptom categories, including fatigue, stomach pain, nausea, frail, joint pain, muscle cramping, loss of appetite, stomach discomfort, vomiting, stomach bloating and acid reflux (hepatic), tremor, change in walking, change in balance, change in writing, other changes in speech, and dizziness (neurological symptoms), and mood-related disturbances such as anxiety, frustration, irritability, depression, frequent up and down in mood, and apathy (psychiatric). The heterogeneity and variety of these symptoms suggests the degree of the burden placed on patients and the extent to which patients’ lives are negatively affected by the disease.

Previously—as indicated by the TLR—research has tended to focus on the neuropsychiatric aspects of the WD presentation in patients with WD, all of which may have a substantial negative impact on patients’ lives [8, 9, 20]. Indeed, even in discussions with a hepatology expert, we found that few hepatic symptoms were considered of central importance relative to the neuropsychiatric features associated with the disease. However, hepatic symptoms are reported by patients in the current research to be both prevalent in their WD journey and highly bothersome. These results suggest that even hepatologists may underestimate the breadth and depth of the impact of hepatic symptoms on patients’ and their daily lives.

In line with current understanding of WD, the CE interviews also suggest that there are 2 distinct group of patients with WD: those with predominant hepatic symptom expression and those with predominant neurological presentation [16, 18]. Our results add to this literature by showing that in both groups these symptoms do not appear in isolation. That is, those who experience primarily hepatic symptoms can also experience psychiatric symptoms that contribute to their patient experience with WD. Furthermore, those who experience neurological symptoms also have a broader range of symptoms (including both hepatic and psychiatric symptoms) that contributes to their patient experience with WD. Using this patient-centric approach therefore helps to understand these known subgroups at a much richer level that shows not only the differential role of neurological symptoms (present in some but not all patients with WD), but also the importance of hepatic symptoms for *all* patients in their experience with WD. Taken together, these results illustrate a more nuanced, comprehensive, and complex picture of the patient disease journey that informs treatment and disease management. In doing so, those patients with a predominant hepatic profile may benefit from discussion and management of psychiatric symptoms to improve how they feel and function in their everyday lives. Likewise, those with a predominant neurological profile may benefit from a level of care that includes attention to neurological, psychiatric, and hepatic symptoms.

By establishing the concepts that are most relevant to patients with WD as well as the differences between the 2 patient groups, this study provides guidance for clinicians to facilitate more effective and meaningful communications with patients compared to dialogues that are based on present knowledge of the WD patient experience. For instance, while the literature conveys the impression that neurological symptoms are the most bothersome to patients, the current research found that 61% of hepatic symptoms were ranked with a bothersome score of at least 5 out of 10 by patients. Clinicians may therefore find it especially valuable to explore the impact of hepatic symptoms on the WD patient experience in their management of these patients.

This study adds to recently published work that presented the results of an online survey conducted with patients with WD and also found the heterogenous nature of WD as well as the wide variety of impacts experienced by patients that effect their quality of life [21]. Unlike the survey method, our research involved qualitative interviews that allowed for spontaneous discussion about the patients’ signs, symptoms, and impacts in their own words. The result of these discussions is a richer patient-centered perspective of what it feels like to live with WD and how the disease impacts the everyday lives of patients.

These findings also have implications for assessing treatment benefit in the context of clinical trials to support endpoint decisions relating to what is most relevant and meaningful to patients. Inclusion of endpoints that focus on neurological symptoms may only be relevant to a subset of patients with WD with these symptoms, whereas endpoints that assess both hepatic and psychiatric symptoms may be used to measure treatment benefit more broadly. These findings may ultimately support the development of meaningful and relevant treatments for WD that address the signs, symptoms and impacts that matter most to patients.

This study has several potential limitations. There is the possibility of selection bias in the patient sample; patients actively volunteered to participate in the research, rather than being approached for recruitment. The resultant study population was small and comprised of adults over the age of 18 years, predominantly female and US-based. Nevertheless, despite WD being a rare disease and the difficulties in patient recruitment for diseases with low prevalence, the sample size (n = 11) is aligned with that of many other studies. In addition, the findings suggest that the interviewed patients covered different disease presentations, disease duration, and comorbidities, allowing for more generalizability of the results. Furthermore, only one caregiver participated in the interviews.

Regarding the clinician sample, although all practiced in the US, these 3 clinicians are considered to be leading experts in WD offering a global clinical perspective, as they routinely treat patients from all around the world. Finally, all patient screeners were completed online, and as barriers to internet access such as socioeconomic status, age, and educational background exist, this may have also limited the generalizability of these findings to larger populations of patients with WD without access to the internet.

The main strengths of this work are that it involves the use of multiple streams of research, including a TLR that included qualitative and quantitative studies, clinician interviews, and patient CE interviews. A further important feature of this work is that the study included patients with a very large range of disease duration (from 5 to 55 years since diagnosis), which allowed for coverage of concepts that are important to patients across the full disease course. This work is, to our knowledge, the first effort to use patient CE interviews to develop a conceptual model for WD. By augmenting established insights from the literature with discussions with expert clinicians, and ultimately with the patients themselves, we can begin to bring a fuller understanding of the patient experience of WD to light. Through the use of CE in particular, patients described their disease and the impact it has on their quality of life in their own words, allowing for a more accurate perspective of how patients feel and function in their everyday lives with this disease. This is integral for understanding and characterizing the WD patient population in a way that was not previously understood, and for identifying the treatment outcomes that matter the most to patients.

## Conclusions

This work establishes a new understanding of what really matters to patients who experience symptoms of WD and how burdensome the different aspects of their condition can be over the course of their journey with this disease. It demonstrates that the hepatic symptoms experienced by many patients disrupt their lives and should be considered when treating these patients. Additionally, it defines subgroups of patients (those with hepatic as well as neurological and psychiatric signs/symptoms, and those without neurological signs/symptoms) that may be key for generating productive dialogue with each group of patients in clinical practice as well as for developing successful therapies for these 2 groups through clinical trials.

## Supplementary Information

**Additional file 1:** Supplemental material to support the methods and results.

## Data Availability

The datasets analyzed for the current study are available on reasonable request.
