# Supplementary material for: The patient experience of Wilson disease: a conceptual model based on qualitative research
Source: Orphanet J Rare Dis. 2021 Oct 19;16:437. doi: 10.1186/s13023-021-02059-x (PMC8525030; doi:10.1186/s13023-021-02059-x)
Supplement: Supplementary file 1 — Additional file 1: Supplemental material to support the methods and results. [file 13023_2021_2059_MOESM1_ESM.docx]

# Supplemental information

Table S1: Literature review results

| 1 | Bandmann O, Weiss KH, Kaler SG. Wilson's disease and other neurological copper disorders. Lancet Neurol. 2015 Jan;14(1):103-13. |
| --- | --- |
| 2 | European Association for Study of Liver. EASL Clinical Practice Guidelines: Wilson's disease. J Hepatol. 2012 Mar;56(3):671-85. |
| 3 | Dzieżyc K, Litwin T, Chabik G, Członkowska A. Frequencies of initial gait disturbances and falls in 100 Wilson's disease patients. Gait & Posture. 2015 Oct;42(4):601-603. |
| 4 | Trocello JM, Osmani K, Pernon M, Chevaillier G, de Brugière C, Remy P, Wenisch E, Cousin C, Girardot-Tinant N, Woimant F. Hypersialorrhea in Wilson's Disease. Dysphagia. 2015 Oct;30(5):489-95. |
| 5 | Tribl GG, Bor-Seng-Shu E, Trindade MC, Lucato LT, Teixeira MJ, Barbosa ER. Wilson's disease presenting as rapid eye movement sleep behavior disorder: a possible window to early treatment. Arq Neuropsiquiatr. 2014 Sep;72(9):653-8. |
| 6 | Biswas S, Paul N, Das SK. Nonmotor Manifestations of Wilson's Disease. Int Rev Neurobiol. 2017;134:1443-1459. |
| 7 | Lorincz MT. Neurologic Wilson's disease. Ann N Y Acad Sci. 2010 Jan;1184:173-87. |
| 8 | Dalvi A. Wilson's disease: neurological and psychiatric manifestations. Dis Mon. 2014 Sep;60(9):460-4. |
| 9 | Litwin T, Dzieżyc K, Karliński M, et al. Early neurological worsening in patients with Wilson's disease. Journal of the Neurological Sciences. 2015 Aug;355(1-2):162-167. |
| 10 | Iwański S, Seniów J, Leśniak M, Litwin T, Członkowska A. Diverse attention deficits in patients with neurologically symptomatic and asymptomatic Wilson's disease. Neuropsychology. 2015 Jan;29(1):25-30. |
| 11 | Lin L, Wang D, Ding N, Zheng C. Hepatic Manifestations in Wilson's Disease: Report of 110 Cases. Hepatogastroenterology. 2015 May;62(139):657-60. |
| 12 | Dalvi A, Padmanaban M. Wilson's disease: etiology, diagnosis, and treatment. Dis Mon. 2014 Sep;60(9):450-9. |
| 13 | Trindade MC, Bittencourt T, Lorenzi-Filho G, Alves RC, de Andrade DC, Fonoff ET, Bor-Seng-Shu E, Machado AA, Teixeira MJ, Barbosa ER, Tribl GG. Restless legs syndrome in Wilson's disease: frequency, characteristics, and mimics. Acta Neurol Scand. 2017 Feb;135(2):211-218. |
| 14 | Mura G, Zimbrean PC, Demelia L, Carta MG. Psychiatric comorbidity in Wilson's disease. Int Rev Psychiatry. 2017 Oct;29(5):445-462. |
| 15 | Zimbrean PC, Schilsky ML. Psychiatric aspects of Wilson disease: a review. Gen Hosp Psychiatry. 2014 Jan-Feb;36(1):53-62. |
| 16 | Przybyłkowski A, Gromadzka G, Chabik G, Wierzchowska A, Litwin T, Członkowska A. Liver cirrhosis in patients newly diagnosed with neurological phenotype of Wilson's disease. Funct Neurol. 2014 Jan-Mar;29(1):23-9. |
| 17 | Członkowska A, Litwin T, Dzieżyc K, Karliński M, Bring J, Bjartmar C. Characteristics of a newly diagnosed Polish cohort of patients with neurological manifestations of Wilson disease evaluated with the Unified Wilson's Disease Rating Scale. BMC Neurol. 2018 Apr 5;18(1):34. |
| 18 | Popević, M., Kisić, G., Đukić, M., & Bulat, P. Work Ability Assessment in a Patient with Wilson's Disease. Archives of Industrial Hygiene and Toxicology. 2011 June 24; 62(2), 163-167. |
| 19 | Svetel M, Pekmezović T, Tomić A, Kresojević N, Potrebić A, Ješić R, Kostić VS. Quality of life in patients with treated and clinically stable Wilson's disease. Mov Disord. 2011 Jul;26(8):1503-8. |
| 20 | Svetel M, Potrebić A, Pekmezović T, Tomić A, Kresojević N, Jesić R, Dragasević N, Kostić VS. Neuropsychiatric aspects of treated Wilson's disease. Parkinsonism Relat Disord. 2009 Dec;15(10):772-5. |
| 21 | Bem RS, Muzzillo DA, Deguti MM, Barbosa ER, Werneck LC, Teive HA. Wilson's disease in southern Brazil: a 40-year follow-up study. Clinics (Sao Paulo). 2011;66(3):411-6. |
| 22 | Woimant F, Djebrani-Oussedik N, Collet C, Girardot N, Poujois A. The hidden face of Wilson's disease. Rev Neurol (Paris). 2018 Nov;174(9):589-596. |
| 23 | Volpert HM, Pfeiffenberger J, Gröner JB, Stremmel W, Gotthardt DN, Schäfer M, Weiss KH, Weiler M. Comparative assessment of clinical rating scales in Wilson's disease. BMC Neurol. 2017 Jul 21;17(1):140. |
| 24 | Hedera P. Wilson's disease: A master of disguise. Parkinsonism Relat Disord. 2019 Feb;59:140-145. |
| 25 | Cochen De Cock V, Girardot-Tinant N, Woimant F, Poujois A. Sleep Abnormalities in Wilson's Disease. Curr Treat Options Neurol. 2018 Sep 27;20(11):46. |
| 26 | Litwin T, Dusek P, Szafrański T, Dzieżyc K, Członkowska A, Rybakowski JK. Psychiatric manifestations in Wilson's disease: possibilities and difficulties for treatment. Ther Adv Psychopharmacol. 2018 Jul;8(7):199-211. |
| 27 | Poujois A, Woimant F. Wilson's disease: A 2017 update. Clin Res Hepatol Gastroenterol. 2018 Dec;42(6):512-520. |
| 28 | Ferenci P, Członkowska A, Merle U, Ferenc S, Gromadzka G, Yurdaydin C, Vogel W, Bruha R, Schmidt HT, Stremmel W. Late-onset Wilson's disease. Gastroenterology. 2007 Apr;132(4):1294-8. |
| 29 | Carta M, Mura G, Sorbello O, Farina G, Demelia L. Quality of Life and Psychiatric Symptoms in Wilson's Disease: the Relevance of Bipolar Disorders. Clin Pract Epidemiol Ment Health. 2012;8:102-9. |
| 30 | Soltanzadeh A, Soltanzadeh P, Nafissi S, Ghorbani A, Sikaroodi H, Lotfi J. Wilson's disease: a great masquerader. Eur Neurol. 2007;57(2):80-5. |

Table S2: Clinician practice characteristics

| Interview Sequence | Clinical Specialty | Experience (years) | Practice setting | Role in WD treatment | WD patients/year |
| --- | --- | --- | --- | --- | --- |
| 1 | Neurology | 20 | Academic Medical Centers | Consultation during initial diagnostic work-up; follow-up care of WD patients with neurological symptoms | 48 |
| 2 | Psychiatry | 12 | Academic Medical Centers | Consultation during initial diagnostic work-up; follow-up care of WD patients with psychiatric/behavioral symptoms | 30 |
| 3 | Hepatology | 32 | Academic Medical Centers | Consultation during initial diagnostic work-up; management of acute and maintenance therapy; follow-up care of hepatic symptoms | 120 |

Table S3: Patient selection criteria

| Inclusion Criteria   - Patient has a clinical diagnosis of WD - Patient is able to provide a confirmation of diagnosis from their clinician or clinician’s office^*^ - Patient currently (i.e., within the last 4 months) experiences symptoms due to their WD or the treatment they are receiving for WD - Patient is 12 years of age or older in the US; 18 years of age or older in the European Union - Patient/caregiver speaks English in the US - Patients/caregiver speaks English or their country’s national language in the EU countries - Patient is willing, able, and capable to provide informed consent/assent to participate in the research (note: parent/guardian consent is required if the patient is less than 18 years old) - Patient/caregiver is willing and able to participate in a 90- to 150-minute telephone interview to discuss signs, symptoms, and impacts related to the patient experience with WD - [US ONLY] Reside in any state in the US (Puerto Rico is excluded) |
| --- |
| Exclusion Criteria   - A mental disability, severe cognitive impairment, or significant mental illness, legal incapacity or limited legal capacity or any other lack of fitness, which, in the opinion of the interview scheduler or interviewer, would preclude the participant’s participation in or ability to complete the study - Unwilling to allow physician or clinical staff verification of health records - Patient has liver failure - Patient has end-stage renal disease on dialysis - Patient has prior/current tetrathiomolybdate use |

*Confirmation of diagnosis forms were collected on a best-effort basis

Table S4: Signs/Symptoms saturation assessment

| **Concept** | **Wave 1** | **Wave 2** | **Wave 3** |
| --- | --- | --- | --- |
| **Hepatic** |  |  |  |
| Yellow skin (jaundice) | X |  |  |
| Vomiting | X |  |  |
| Nausea | X |  |  |
| Diarrhea | X |  |  |
| Acid Reflux | X |  |  |
| Swelling/fluid retention | X |  |  |
| Stomach pain | X |  |  |
| Stomach discomfort | X |  |  |
| Stomach bloating | X |  |  |
| Loss of appetite | X |  |  |
| Weight loss | X |  |  |
| Spider veins (small damaged veins visible on the surface of the legs or face) | X |  |  |
| Tendency to bleed easily | X |  |  |
| Easily bruised | X |  |  |
| Increased nose bleeds | X |  |  |
| Frail (fragile, physically vulnerable, low energy levels) | X |  |  |
| Fatigue (extreme tiredness, low energy levels) | X |  |  |
| Night sweats | X |  |  |
| Shortness of breath | X |  |  |
| Enlarged/swollen liver | X |  |  |
| Anemia | X |  |  |
| Portal vein hypertension | X |  |  |
| Hair loss |  | X |  |
| Itchiness/Dry skin |  | X |  |
| Muscle cramping (including soreness/sensitivity) | X |  |  |
| Joint pain | X |  |  |
| Joint stiffness | X |  |  |
| Joint swelling | X |  |  |
| Green brown/gold rings around eyes | X |  |  |
| Light sensitivity | X |  |  |
| Stunted growth (endocrine disturbances) | X |  |  |
| **Neurological** |  |  |  |
| Slurred speech |  |  | X |
| Other change in speech (vocal tremor, stutter, word selection, word misuse) | X |  |  |
| Drooling |  | X |  |
| Tremor | X |  |  |
| Change in writing (tinier, messier than usual) | X |  |  |
| Change in walking | X |  |  |
| Change in balance | X |  |  |
| Bad posture | X |  |  |
| Involuntary muscle movement | X |  |  |
| Restless legs | X |  |  |
| Dystonia (twitching leading to twisting motion) | X |  |  |
| Difficulty swallowing | X |  |  |
| Headache | X |  |  |
| Dizziness | X |  |  |
| Vertigo |  |  | X |
| Ringing in the ear |  | X |  |
| Fainting |  |  | X |
| Difficulty solving problems |  | X |  |
| Difficulty with decision making |  | X |  |
| **Psychiatric** |  |  |  |
| Anxiety (states of fear) | X |  |  |
| Depression (sad or blue) | X |  |  |
| Changes in thinking skills (feeling slowed down, forgetful, slow processing) |  | X |  |
| Changes in attention (trouble focusing; easily distracted) |  | X |  |
| Frequent up and down in mood | X |  |  |
| Apathy (feeling disengaged, feeling like don’t care about anything) | X |  |  |
| Hyperactivity (cannot sit still, restless) | X |  |  |
| Irritability | X |  |  |
| Impulsive | X |  |  |
| Disinhibited (difficult to control behavior or speech) |  | X |  |
| Anger (rage) | X |  |  |
| Frustration | X |  |  |
| Night terror/vivid dreams |  | X |  |

Table S5: Impacts saturation assessment

| **Concept** | **Wave 1** | **Wave 2** | **Wave 3** |
| --- | --- | --- | --- |
| Physical manifestation of emotional distress | X |  |  |
| Limit in physical function | X |  |  |
| Sleep disturbance | X |  |  |
| Difficulty writing | X |  |  |
| Limit in usual activity (e.g., eating, bathing, dressing, toileting) |  |  | X |
| Excessive daytime sleep | X |  |  |
| Intentional self-harm | X |  |  |
| Changes in self-perception | X |  |  |
| Worried about the future (e.g., passing to kids) | X |  |  |
| Embarrassed | X |  |  |
| Made fun of/ridiculed by others | X |  |  |
| Feeling scared | X |  |  |
| Feeling sad | X |  |  |
| Worried about how perceived by others | X |  |  |
| Difficulty planning your day | X |  |  |
| Impact on ability to work (on disability, reduced work performance) | X |  |  |
| Impact on school performance | X |  |  |
| Impact on family life | X |  |  |
| Impact on social life | X |  |  |
| Time burden (e.g., going to Dr. appt/treatment regimen) | X |  |  |
| Financial burden | X |  |  |

Table S6: Signs and Symptoms – number of patients having experienced and average peak bother rating

| **Concept** | **# of patients ever mentioning** | **Average peak bother – ever experienced** |
| --- | --- | --- |
| **Hepatic** |  |  |
| Fatigue (extreme tiredness, low energy levels) | 11 | 8.5 |
| Nausea | 8 | 8 |
| Stomach pain | 8 | 8 |
| Frail (fragile, physically vulnerable, low energy levels) | 7 | 7.7 |
| Joint pain | 7 | 9 |
| Muscle cramping (including soreness/sensitivity) | 6 | 7 |
| Stomach discomfort | 6 | 6.2 |
| Weight loss | 6 | 4.2 |
| Loss of appetite | 6 | 7.6 |
| Vomiting | 5 | 8 |
| Stomach bloating | 5 | 7.5 |
| Acid Reflux | 5 | 6.6 |
| Easily bruised | 5 | 3.6 |
| Increased nose bleeds | 4 | 7.3 |
| Shortness of breath | 4 | 6 |
| Joint stiffness | 4 | 5.5 |
| Diarrhea | 4 | 5.3 |
| Spider veins (small damaged veins visible on the surface of the legs or face) | 4 | 5 |
| Swelling/fluid retention | 4 | 4.5 |
| Enlarged/swollen liver | 4 | 3 |
| Green brown/gold rings around eyes | 4 | 1.3 |
| Yellow skin (jaundice) | 3 | 6.7 |
| Night sweats | 3 | 7.3 |
| Itchiness/Dry skin | 3 | 5.3 |
| Tendency to bleed easily | 3 | 3.3 |
| Hair loss | 2 | 8.5 |
| Anemia | 2 | 7.5 |
| Portal vein hypertension | 1 | 8 |
| Light sensitivity | 1 | 2 |
| Stunted growth (endocrine disturbances) | 1 | 0 |
| Joint swelling | 1 |  |
| Lower extremity pain related to swelling (e.g., feet pain) | 0 | -- |
| Skin rash | 0 | -- |
| Blurred vision | 0 | -- |
| **Neurological** |  |  |
| Change in walking | 7 | 7.2 |
| Change in balance | 6 | 8.3 |
| Other change in speech (vocal tremor, stutter, word selection, word misuse) | 5 | 7.4 |
| Change in writing (tinier, messier than usual) | 5 | 7.4 |
| Tremor | 5 | 6.4 |
| Dizziness | 5 | 6 |
| Ringing in the ear | 4 | 7.8 |
| Difficulty swallowing | 4 | 8.5 |
| Restless legs | 3 | 8.7 |
| Vertigo | 3 | 8.7 |
| Headache | 3 | 6.3 |
| Bad posture | 3 | 0.3 |
| Slurred speech | 2 | 9 |
| Drooling | 2 | 8.5 |
| Dystonia (twitching leading to twisting motion) | 2 | 5 |
| Difficulty with decision making | 2 | 5.5 |
| Involuntary muscle movement | 2 | 5.5 |
| Fainting | 1 | 10 |
| Difficulty solving problems | 1 | 9 |
| Changes in facial expression | 0 | -- |
| Asymmetry of face | 0 | -- |
| Unable to walk/unable to talk | 0 | -- |
| Difficulty eating | 0 | -- |
| Seizures | 0 | -- |
| Numbness in jaw | 0 | -- |
| **Psychiatric** |  |  |
| Anxiety (states of fear) | 8 | 7.5 |
| Irritability | 7 | 5.6 |
| Frustration | 7 | 6 |
| Depression (sad or blue) | 6 | 8.5 |
| Night terrors/vivid dreams | 6 | 3.8 |
| Frequent up and down in mood | 5 | 6.2 |
| Apathy (feeling disengaged, feeling like don’t care about anything) | 5 | 6 |
| Impulsive | 5 | 3.2 |
| Anger (rage) | 4 | 8.5 |
| Hyperactivity (cannot sit still, restless) | 4 | 4.5 |
| Changes in thinking skills (feeling slowed down, forgetful, slow processing) | 3 | 10 |
| Changes in attention (trouble focusing; easily distracted) | 3 | 8.7 |
| Disinhibited (difficult to control behavior or speech) | 1 | 5 |
| Psychotic episode (hearing voices that no one else hears, seeing things that are not really there) | 0 | -- |
| Mania | 0 | -- |

: not asked | -- : not reported

Table S7: Impacts – number of patients having experienced and average peak bother rating

| **Concept** | **# of patients ever mentioning** | **Average peak bother – ever experienced** |
| --- | --- | --- |
| Worried about the future (e.g., passing to kids) | 10 | 6.1 |
| Sleep disturbance | 10 | 6.2 |
| Feeling scared | 9 | 6 |
| Limit in physical function | 8 | 6.9 |
| Impact on school performance | 7 | 7 |
| Made fun of/ridiculed by others | 7 | 7.6 |
| Impact on ability to work (on disability, reduced work performance) | 7 | 7 |
| Time burden (e.g., going to Dr. appt/treatment regimen) | 7 | 7.2 |
| Impact on social life | 7 | 7 |
| Feeling sad | 6 | 4.2 |
| Excessive daytime sleep | 6 | 7.3 |
| Financial burden | 5 | 7.4 |
| Embarrassed | 5 | 7.2 |
| Worried about how perceived by others | 5 | 8 |
| Difficulty writing | 5 | 5.8 |
| Impact on family life | 5 | 5.8 |
| Changes in self-perception | 4 | 6.3 |
| Difficulty planning your day | 4 | 6.3 |
| Physical manifestation of emotional distress | 2 | 4.5 |
| Limit in usual activity (e.g., eating, bathing, dressing, toileting) | 1 | 9 |
| Intentional self-harm | 1 | 1 |
| Inability to walk/wheelchair bound | 0 | -- |
| Alcohol/abuse | 0 | -- |

: not asked | -- : not reported

Figure S1: Preliminary conceptual model based on literature review


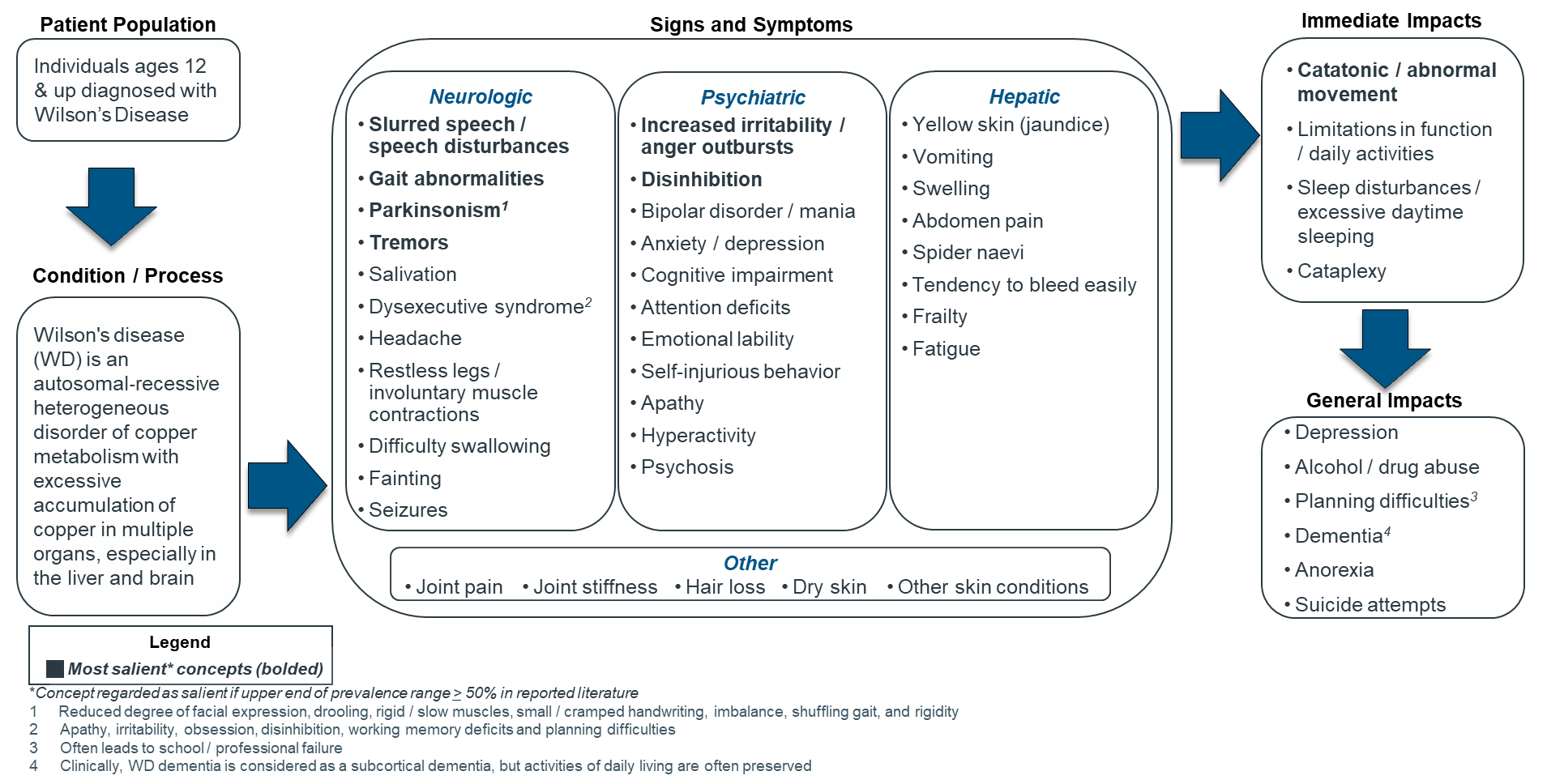


Figure S2: Interim conceptual model based on clinician interviews
